# Long-term exposure to ambient air pollution and risk of incident acute myocardial infarction in a nationwide register-based cohort study

**DOI:** 10.1007/s00420-025-02198-9

**Published:** 2026-01-16

**Authors:** Nikoline Leo Fleischer, Esben Meulengracht Flachs, Matthias Ketzel, Jørgen Brandt, Jibran Khan, Per Gustavsson, Ingrid Sivesind Mehlum, Morten Böttcher, Camilla Sandal Sejbaek, Jens Peter Bonde, Regitze Sølling Wils

**Affiliations:** 1 Department of Occupational and Environmental Medicine, Copenhagen University Hospital – Bispebjerg and Frederiksberg, Copenhagen, Denmark; 2 Department of Environmental Science, Aarhus University, Roskilde, Denmark; 3 Danish Big Data Centre for Environment and Health (BERTHA), Aarhus University, Roskilde, Denmark; 4 Department of Environment and Biological Sciences, University of Eastern Finland, Kuopio, Finland; 5 Institute of Environmental Medicine, Karolinska Institutet, Stockholm, Sweden; 6 Department of Public Health, University of Copenhagen, Copenhagen, Denmark; 7 Department of Occupational Medicine and Epidemiology, National Institute of Occupational Health (STAMI), Oslo, Norway; 8 Department of Cardiology, University Clinic for Cardiovascular Research, Gødstrup Hospital, Herning, Denmark; 9 Department of Clinical Medicine, Aarhus University, Aarhus, Denmark

**Keywords:** Ambient air pollution, Acute myocardial infarction, Epidemiology, Cohort studies

## Abstract

**Objective:**

The aim of this study was to investigate the association between long-term exposure to ambient air pollution at the residential address and the risk of acute myocardial infarction (AMI) in a younger working population.

**Methods:**

The study population included all Danish residents aged 35–50 in 1995 with employment and no previous diagnosis of AMI. Information on AMI was obtained through national registries. We estimated the exposure to fine particulate matter (PM_2.5_), nitrogen dioxide (NO_2_), elemental carbon (EC) and primary organic aerosols (POA) from 1979 onwards based on the Danish integrated multi-scale air pollution modeling system. Poisson regression models were used to estimate incidence rate ratios (IRR) with 95% confidence intervals (CI) of AMI.

**Results:**

903,415 individuals were included in this cohort, covering almost 20 million person-years of follow-up between 1996 and 2018. In total 35,511 developed AMI. Our main analyses showed a clear exposure-response relationship between cumulative exposure to each of the air pollutants and incident AMI. Exposure was categorized into quartiles (Q1–Q4), with Q1 as reference. The most pronounced association was observed for PM_2.5_ with IRRs of 1.08 [95% CI 1.04,1.13], 1.14 [95% CI 1.09,1.20] and 1.24 [95% CI 1.18,1.31] in Q2–Q4 in model 4 (fully adjusted). The associations were observed for both sexes and across age groups.

**Conclusion:**

Our findings suggest that long-term exposure to PM_2.5_, NO_2_, EC, and POA are associated with an increased risk of incident AMI, also pertaining to the younger population (< 55 years).

**Supplementary Information:**

The online version contains supplementary material available at 10.1007/s00420-025-02198-9.

## Introduction

Ambient air pollution is a major environmental risk factor for mortality and morbidity and is estimated to account for approximately 6.7 million deaths per year globally (Fuller et al. 2022; WHOa 2022). Exposure to air pollution has been associated with an increased risk of cardiovascular disease (Brook et al. 2004; Franklin et al. 2015). Evidence from mechanistic studies suggests that air pollution can cause pulmonary inflammation, induce an imbalance of the autonomic nervous system, and a direct translocation of pollution particles into the bloodstream (Brook et al. 2010; Miller and Newby 2019). These pathways may result in systemic inflammation, oxidative stress, vasoconstriction, formation of atherosclerosis and increase thrombosis formation, potentially leading to cardiovascular disease (Brook et al. 2010; Kaufman et al. 2016; Miller and Newby 2019). Given that ischemic heart disease (IHD), including acute myocardial infarction (AMI), is the most prevalent cardiovascular disease, and the leading cause of death globally (Khan et al. 2020; WHOb, n.d.), it is essential to examine the long-term health impacts of ambient air pollution on the development of IHD.

Previous studies on air pollution have mainly focused on exposure to fine particulate matter (PM_2.5_), with an aerodynamic diameter <2.5 μm (Brook et al. 2010). Several studies have reported an association between long-term exposure to PM_2.5_ and increased risk of IHD (Bai et al. 2019a, b; Chen and Hoek 2020; Crouse et al. 2012; Hayes et al. 2020; Kim et al. 2017; Pope et al. 2004), whereas other studies did not find this association (Atkinson et al. 2013; Beelen et al. 2014; Carlsen et al. 2022; Downward et al. 2018; Gan et al. 2011; Ljungman et al. 2019). Fewer studies have examined long-term exposure to other indicators of ambient air pollution such as nitrogen dioxide (NO_2_), elemental carbon (EC), and primary organic aerosols (POA) in relation to IHD, and the findings are ambiguous (Carlsen et al. 2022; Cesaroni et al. 2014; Gan et al. 2011; Roswall et al. 2017; Yang et al. 2020). Therefore, more research is warranted regarding the health impacts of long-term exposure to PM_2.5_, NO_2_, EC, and POA, respectively, and risk of IHD.

A meta-analysis demonstrated a statistically significant association between PM_2.5_ and IHD mortality, but not with incident AMI, including both fatal and non-fatal cases (Alexeeff et al. 2021), which is consistent with findings from other studies (Cramer et al. 2020; Lipsett et al. 2011; Miller et al. 2007; Puett et al. 2009). Therefore, the authors of the meta-analysis warranted more studies on non-fatal outcomes such as incident AMI, where the number of events would be less sensitive to especially the age distribution in the population.

Recent studies have found a significantly increased risk of incident AMI in relation to PM_2.5_ (Alexeeff et al. 2023; Olaniyan et al. 2022; Poulsen et al. 2023; Wei et al. 2024). However, many epidemiological studies include a population only consisting of elderly, thus comprising individuals highly susceptible to IHD due to age and/or comorbidity (Miller et al. 2007; Poulsen et al. 2023; Roswall et al. 2017; Wei et al. 2024). Only a few studies have investigated the association in a younger population (Alexeeff et al. 2023). Therefore, more studies on long-term exposure to air pollution and the risk of AMI in younger individuals are warranted. Although the absolute risk is lower in the younger population, the potential impact of incident AMI on quality-adjusted life years, life expectancy and general health could be substantial.

In the present study, we therefore aim to examine the association between multiple indicators of ambient air pollution at the residential address and the risk of incident AMI in a somewhat younger population (35–50 years of age in 1995), without any history of AMI and gainfully employed in 1995. Registries on occupational history enable us, through the use of a quantitative job exposure matrix (JEM), to adjust for occupational exposure to diesel engine exhaust (DEE), which was found to be a risk factor for AMI in a recent study (Wils et al. 2025), and has not been taken into account in previous studies.

## Methods

### Study population

The DOC*X Dust cohort, which is a subpopulation of the DOC*X cohort described previously (Flachs et al. 2019), consists of data collected from 1976 to 2018, providing comprehensive annual information on occupation, health, and demographics of the entire Danish workforce. Data on air pollution was available for the period 1979 to 2018. At baseline, on January 1st, 1995, 1,255,436 members of the population were aged 35–50 years (ranging from 19 to 34 years in 1979, and from 58 to 73 years in 2018). We excluded individuals who did not reside in Denmark at start of follow-up on January 1st, 1996 (*n* = 130,251), and individuals who had experienced an AMI (ICD-10 code I21.0-I21.9 or ICD-8 code 410) prior to start of follow-up (*n* = 3614). Moreover, as control for potential confounding from occupational exposure to DEE was considered an important novel strength of our study, we also excluded members of the workforce with no or very limited data on occupational history. That is, individuals without DISCO-88 (The Danish version of ISCO-88) job codes (Flachs et al. 2019), with a minimum of two digits at baseline and at least one four-digit job code within the preceding 5 years (*n* = 16,889), individuals employed in the military (*n* = 4909), individuals who were completely absent from work between 1992 and 1995 (*n* = 5482), and individuals who had not been living a total of 5 years in Denmark between 1976 and 1993 (*n* = 3869). The study population therefore includes 903,415 persons who were followed until occurrence of AMI, death, emigration, or end of follow-up on December 31st, 2018, whichever came first.

### Exposure assessment

In this study, we selected PM_2.5_, NO_2_, EC and POA as indicators of ambient air pollution. All four indicators are inextricably linked, e.g., EC and POA are a subset of PM_2.5_, and NO_2_ is linked to PM_2.5_ through the formation of secondary pollutants from activation of NO_2_ to particles measured as PM_2.5_. All four indicators have common sources such as road traffic, industry emissions, and other combustion processes (IARC Working Group on the Evaluation of Carcinogenic Risks to Humans 2016; WHOc 2021).

The exposure to ambient air pollution at the residential address was estimated with the integrated air pollution modeling system (DEHM/UBM/AirGIS), which is a fully deterministic Chemistry-Transport-Model (CTM) system (http://au.dk/AirGIS/), based on emissions, meteorology, atmospheric physics, and chemistry (Jensen et al. 2017; Ketzel et al. 2011; Khan et al. 2019). The system consists of a meteorological model and a coupling of three air pollution models, covering different scales: (1) the Danish Eulerian Hemispheric Model (DEHM), which models air pollution in the Northern Hemisphere, in order to account for intercontinental transport of air pollution. The model is set up with 4 nested domains with different resolution, where the fourth domain covers Denmark with a spatial resolution of 5.6 km × 5.6 km (Brandt et al. 2012; Christensen 1997; Frohn et al. 2021); (2) the Urban Background Model (UBM), which uses output from the DEHM and the meteorological model as well as detailed Danish emissions to give concentrations with a 1 km × 1 km resolution over Denmark (Brandt et al. 2001, 2003; Frohn et al. 2022); and (3) the Operational Street Pollution Model (OSPM), which uses results from the UBM, meteorology, street traffic emissions, and street geometry as input and provides concentrations for every street with more than 500 vehicles/day (Hvidtfeldt et al. 2018; Ketzel et al. 2012, 2021). The DEHM/UBM/AirGIS modelling system provides estimates of air concentrations of a range of air pollutants from 1979 to 2018 with a one-hour time resolution, and is continuously developed further and validated with all available measurements from all measuring stations of the Danish Monitoring Program and in Europe (Ellermann et al. 2024).

An evaluation shows high correlations between air pollution concentrations (PM_2.5_, NO_2_, EC and POA) that were modeled with DEHM/UBM/AirGIS and independent field measurements. The correlation coefficients for modelled and measured values were between 0.87 and 0.99 for annual means, between 0.73 and 0.93 for monthly means, and between 0.68 and 0.87 for daily means, when considering the combined spatial and temporal performance for all available measurements (Ellermann et al. 2024; Khan et al. 2019).

Regarding individuals who had an unidentified address at some point, the mean exposure level of the entire population the given year was inserted. Missing addresses accounted for 0.69% of all person years 1979–2017.

### Outcome

Information on first-time incident AMI (ICD-10 code I21.0-I21.9) was obtained through the Danish National Patient Register (DNPR) during the follow-up period from 1996 to 2018 using the unique identification numbers from the Central Person Register. Additionally, non-hospitalized cases of fatal AMI were assessed through the Danish Registry of Causes of Death. The validity of diagnosing first-time AMI in the DNPR has been examined by cross-referencing the registry diagnosis with medical records. The resulting positive predictive value was consistently above 90% across studies (Madsen 2003; Thygesen et al. 2011).

### Covariates

Potential confounders were selected prior to the study based on a directed acyclic graph (DAG) (Fig. S1). We acquired annually updated information on civil status (partner; no partner), highest obtained education level (lower secondary; upper secondary; short tertiary; medium tertiary; long tertiary), and income (yearly quartiles) from Statistics Denmark. Furthermore, body mass index (BMI) and smoking probability were assessed using Danish lifestyle-JEMs taking job code, age, sex, and calendar year into account. A historical, quantitative JEM based on Swedish working conditions was also used to estimate yearly occupational exposure to DEE across 52 different occupations. In the JEM, EC estimates were used as a proxy since the correlation with particulates in DEE is high. We adjusted DEE estimates by taking absence from work into account. Information on absence from work was provided by the DREAM database from 1991 and onwards (Hjollund et al. 2007).

### Statistical analyses

We used Poisson regression analyses to estimate incidence rate ratios (IRR) with corresponding 95% confidence intervals (CI) for associations between PM_2.5_, NO_2_, EC, and POA, respectively, and incidence of AMI. We chose to perform Poisson regression analyses because our outcome was count-based and given that the model is equivalent to a Cox regression model. The dispersion test indicated no evidence of overdispersion. For each air pollutant, we investigated exposure as cumulated and as recent exposure. For each calendar year, the mean monthly values of air concentrations were summarized throughout the observation period (1979–2017) with a running cumulative value of exposure to air pollution. The cumulated annual exposure was employed to account for the potential adverse health impacts associated with prolonged high exposure to air pollution. To ensure that exposure preceded the event, we lagged each year at risk by 1 year. We categorized the total cumulative exposure from 1996 to 2017 into quartiles (Q1–Q4), with Q1 being our reference. Exposure was categorized into quartiles to examine potential non-linear associations between cumulative exposure to each of the four air pollutants and AMI. In addition, we also investigated recent exposure, defined as the exposure level the preceding year. Recent exposure was divided into quartiles and lagged by 1 year as well. When examining recent exposure, we adjusted for cumulative exposure lagged by 2 years, in order to isolate the effect of recent exposure. Spearman rank correlation coefficients were estimated to assess the correlation between the four cumulative exposures.

We used a stepwise approach to adjust for the identified confounders in our DAG (Fig. S1), which were all lagged by 1 year. Model 1 was minimally adjusted and included age, sex, and calendar year. In model 2, we additionally adjusted for cumulative occupational exposure to DEE. Model 3 encompassed the factors adjusted for in model 1, as well as sociodemographic and lifestyle factors, including education, income, civil status, cumulative years with a likelihood of having a high BMI (≥75th percentile) and cumulative years with a likelihood of being a smoker (≥75th percentile). Lastly, in model 4 we adjusted for all the covariates included in model 1 through 3.

Based on model 4 (fully adjusted), we also assessed the associations between cumulative exposure to each of the four air pollutants, treated as continuous variables, and the risk of AMI per 1000 person-years (PY) for both males and females, using linear splines with knots placed at the 10th, 25th, 50th, 75th, and 90th percentiles. We also performed stratified analyses to address possible effect modification by age and sex. Age was dichotomized into <55 years and ≥55 years.

As PM_2.5_ and NO_2_ are correlated through common emission sources such as traffic or combustion processes (WHOc 2021), supplementary analyses with mutual adjustments for each component were carried out. Additionally, analyses of the associations per interquartile range (IQR) increase for all four indicators of air pollution were conducted for cumulative measures as well. Absolute risk was also estimated for both sexes and the two age groups.

## Results

The study population of 903,415 individuals accumulated to 19,357,326 person-years of follow-up. The total number of AMIs in the follow-up period was 35,511, including pre-hospital deaths due to AMI (accounting for 7% (*n* = 2523)). At baseline in 1995, the distribution of males and females was approximately even, the median age was 41.6 years, with 50% of the study population between the ages of 37.6 and 45.6 years, and 70.4% had completed an upper or lower secondary education as their highest education, while 6.4% had a long tertiary education (Table 1). The majority of the population had a partner (70.7%). At baseline 13.8% was in the fourth quartile for cumulative years of having a BMI above the 75th percentile, based on the lifestyle-JEM, whereas for smoking, the percentage was 5.7%. Spearman’s rank correlation coefficients indicated that the quartiles across all four air pollutants were highly correlated (Table S2). The strongest correlation (0.89) was observed between EC and NO_2_ and the lowest correlation (0.68) was found between EC and PM_2.5_ based on the correlation between the quartiles for each exposure.

**Table 1:** Descriptive characteristics of the DOC*X dust cohort (*N* = 903,415) at baseline (1995)

Baseline characteristics	Total (%)
*Sex*	
Male	454,902 (50.4)
Female	448,513 (49.6)
Age (y)^a^	41.6 (37.6, 45.6)
*Highest education*	
Long tertiary	57,699 (6.4)
Medium tertiary	162,200 (18.0)
Short tertiary	35,723 (4.0)
Upper secondary	405,868 (44.9)
Lower secondary	230,056 (25.5)
Missing	11,869 (1.3)
*Income*	
Highest quartile	214,611 (25.0)
Missing	44,970 (5.0)
*Civil status*	
Partner	638,910 (70.7)
No partner	264,505 (29.3)
*BMI*	
Highest quartile^b^	124,847 (13.8)
Smoking probability	
Highest quartile^b^	51,252 (5.7)
*DEE*	
Ever	264,496 (29.3)
Never	638,913 (70.7)
PM_2.5_^a^	13.5 (12.6, 14.1)
NO_2_^a^	18.6 (15.2, 23.5)
EC^a^	0.8 (0.7, 1.0)
POA^a^	1.5 (1.3, 1.7)

y, year; DEE, diesel engine exhaust; PM_2.5_, fine particulate matter; NO_2_, nitrogen dioxide; EC, elemental carbon; POA, primary organic aerosols

^a^Presented as median (25., 75. percentile)

^b^Presented for 1995 but based on all BMI-values or smoking probabilities 1976–2018

**Fig. 1:**
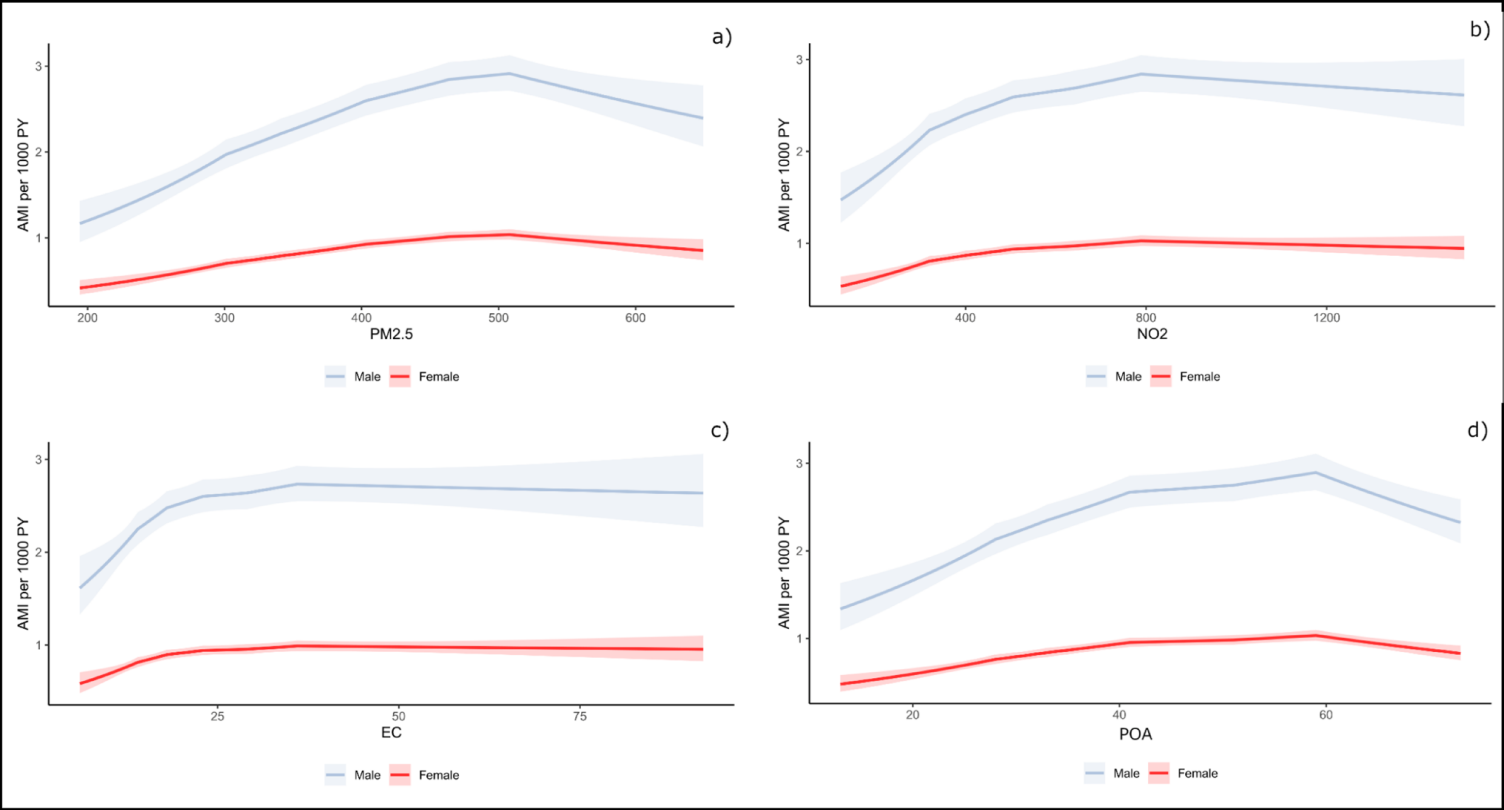
Splines illustrating predicted AMIs per 1000 PY for males and females with 95% CIs for PM_2.5_ (µg/m^3^) (**a**), NO_2_ (µg/m^3^) (**b**), EC (µg/m^3^) (**c**), and POA (µg/m^3^) (**d**). Knots were placed at the 10th, 25th, 50th, 75th, and 90th percentiles. Each spline is based on the following selected categories for each variable: age = 55, education = lower secondary, income = Q1, civil status = partner, BMI = Q2, Smoking = Q1, diesel = never, and year = 2010. The highest 0.01% in each exposure is removed due to extremely high values and as a result, wide CIs

Splines illustrated a non-linear association between cumulative exposure to PM_2.5_, NO_2_, EC, and POA, respectively, and AMI per 1000 PY for both males and females, with an increase followed by a plateau or even a small decline (Fig. 1). Results from all four models showed an exposure-response relationship for the association between cumulative exposure to PM_2.5_, NO_2_, EC, and POA and the risk of AMI across quartiles (Table 2). The most pronounced association was observed for PM_2.5_, with an IRR of 1.24 [95% CI 1.18, 1.31] for incident AMI in Q4 in model 4 (Table 2). However, when adjusting for PM_2.5_ and NO_2_ mutually, higher point estimates of IRR were found for cumulative exposure to NO_2_ than for PM_2.5_ in model 4 (Table S3). The IRRs generally increased slightly when adjusting for additional covariates (Table 2), also illustrated in the estimated IRRs per interquartile range (IQR) (Table S4). Notably, Q4 showed elevated IRRs in model 4 compared to model 1, with an increase from 1.05 [95% CI 1.00, 1.10] to 1.18 [95% CI 1.13, 1.24] for POA (Table 2). For both EC and POA, the IRRs per IQR demonstrated a non-significant association with incident AMI in model 1 opposed to a statistic significant association in model 4 (Table S4). Additional adjustment for occupational exposure to DEE in model 4 compared to model 3, which accounted for socioeconomic status and lifestyle factors, did not alter the estimates (Table 2).

**Table 2:** Associations between cumulative exposure (1979–2017) to PM_2.5_, NO_2_, EC and POA and incidence rate ratios for incident AMI

				Incident AMI			
Exposure (µg/m^3^ y)	Mean	Person-years	Cases	Model 1^a^	Model 2^b^	Model 3^c^	Model 4^d^
				IRR (95% CI)	IRR (95% CI)	IRR (95% CI)	IRR (95% CI)
*Cumulative PM*_2.5_							
Q1 (194.6–340.9)	303.6	4,847,102	5329	1	1	1	1
Q2 (>340.9–402.7)	372.1	4,842,849	7875	1.08 [1.04, 1.13]	1.09 [1.05, 1.13]	1.08 [1.04, 1.13]	1.08 [1.04, 1.13]
Q3 (>402.7–464.2)	433.0	4,837,303	10,166	1.13 [1.07, 1.18]	1.14 [1.09, 1.20]	1.14 [1.09, 1.20]	1.14 [1.09, 1.20]
Q4 (>464.2–1319.7)	505.4	4,830,072	12,141	1.19 [1.13, 1.26]	1.22 [1.15, 1.29]	1.24 [1.17, 1.31]	1.24 [1.18, 1.31]
*Cumulative NO*_2_							
Q1 (123.7–398.5)	326.8	4,845,233	6438	1	1	1	1
Q2 (>398.5–505.2)	452.1	4,841,274	8489	1.06 [1.03, 1.10]	1.07 [1.04, 1.11]	1.08 [1.05, 1.12]	1.08 [1.05, 1.12]
Q3 (>505.2–640.0)	567.0	4,837,732	9701	1.08 [1.05, 1.12]	1.10 [1.07, 1.14]	1.13 [1.09, 1.17]	1.13 [1.09, 1.17]
Q4 (>640.0–2551.3)	795.8	4,833,087	10,883	1.11 [1.07, 1.15]	1.14 [1.10, 1.19]	1.19 [1.15, 1.24]	1.20 [1.15, 1.24]
*Cumulative EC*							
Q1 (6.0–17.9)	14.7	4,844,899	6612	1	1	1	1
Q2 (>17.9–22.8)	20.3	4,840,927	8686	1.06 [1.02, 1.09]	1.07 [1.03, 1.10]	1.08 [1.05, 1.12]	1.08 [1.05, 1.12]
Q3 (>22.8–29.2)	25.7	4,837,663	9750	1.06 [1.03, 1.10]	1.08 [1.05, 1.12]	1.11 [1.07, 1.15]	1.11 [1.08, 1.15]
Q4 (>29.2–815.9)	37.8	4,833,837	10,463	1.06 [1.03, 1.10]	1.10 [1.06, 1.14]	1.14 [1.10, 1.18]	1.14 [1.10, 1.18]
*Cumulative POA*							
Q1 (13.8–32.9)	28.0	4,846,602	5612	1	1	1	1
Q2 (>32.9–41.1)	36.9	4,842,705	7912	1.05 [1.01, 1.09]	1.07 [1.03, 1.11]	1.08 [1.04, 1.12]	1.08 [1.04, 1.12]
Q3 (>41.1–50.7)	45.7	4,836,971	10,349	1.08 [1.03, 1.13]	1.11 [1.06, 1.15]	1.14 [1.10, 1.19]	1.14 [1.10, 1.19]
Q4 (>50.7–94.5)	58.1	4,831,048	11,638	1.05 [1.00, 1.10]	1.10 [1.05, 1.15]	1.18 [1.12, 1.24]	1.18 [1.13, 1.24]

y, year; IRR, incidence rate ratio; CI, confidence interval; AMI, acute myocardial infarction; PM_2.5_, fine particulate matter; NO_2_, nitrogen dioxide; EC, elemental carbon; POA, primary organic aerosols; Q1–Q4, quartile 1–4

^a^Adjusted for age, sex, and calendar year

^b^Model 1 + additional adjustment for occupational exposure to diesel exhaust

^c^Model 1 + additional adjustment for socioeconomic factors, including education and income, civil status, BMI, and smoking

^d^Adjusted for all of the above

The IRRs for recent exposure were lower than for cumulative exposure and increased risk of AMI was only significant in association with PM_2.5_ (Table S5). However, the IRRs for NO_2_ still indicated an exposure-response relationship with increased risk of incident AMI across the quartiles.

Stratified analyses based on model 4 showed an increased relative risk of AMI in association with cumulative exposure to all four air pollutants for both the younger (<55 years) and the older (≥55 years) age group (Table 3). Regarding recent exposure, the IRRs for all four exposures were more pronounced among the age group <55 years, and with fewer significant estimates among the age group ≥55 years (Table S6). Sex-stratified analyses indicated that women may be more susceptible to cumulative exposure to PM_2.5_ in relation to AMI, with an IRR of 1.39 [95% CI 1.23, 1.56] in Q4, compared to an IRR of 1.22 [95% CI 1.14, 1.30] among men (Table 3). However, men and older age groups (≥55 years) still showed higher absolute risks of AMI in association with all four air pollutants compared to women and the younger age group, respectively (Table S7).

**Table 3:** Age- and sex-stratified analyses for cumulative exposure (1979–2017) to PM_2.5_, NO_2_, EC and POA and incidence rate ratios for incident AMI (model 4)

		Incident AMI		
	Male^a^	Female^a^	Age <55^b^	Age ≥55^b^
Exposure	IRR (95% CI)	IRR (95% CI)	IRR (95% CI)	IRR (95% CI)
Person-years (cases)	9,591,629 (27,047)	9,827,707 (8464)	10,359,976 (12,929)	9,059,060 (22,582)
*Cumulative PM*_2.5_				
Q1	1	1	1	1
Q2	1.07 [1.02, 1.11]	1.20 [1.09, 1.31]	1.11 [1.06, 1.17]	1.13 [1.03, 1.24]
Q3	1.11 [1.05, 1.17]	1.32 [1.19, 1.46]	1.14 [1.06, 1.22]	1.20 [1.09, 1.32]
Q4	1.22 [1.14, 1.30]	1.39 [1.23, 1.56]	1.22 [1.10, 1.35]	1.28 [1.16, 1.41]
*Cumulative NO*_2_				
Q1	1	1	1	1
Q2	1.08 [1.04, 1.12]	1.10 [1.02, 1.18]	1.09 [1.04, 1.14]	1.09 [1.03, 1.15]
Q3	1.12 [1.08, 1.17]	1.18 [1.10, 1.27]	1.15 [1.10, 1.21]	1.12 [1.07, 1.18]
Q4	1.20 [1.16, 1.25]	1.20 [1.11, 1.29]	1.22 [1.15, 1.30]	1.17 [1.11, 1.23]
*Cumulative EC*				
Q1	1	1	1	1
Q2	1.08 [1.04, 1.13]	1.08 [1.01, 1.16]	1.09 [1.04, 1.14]	1.08 [1.03, 1.14]
Q3	1.11 [1.07, 1.16]	1.12 [1.04, 1.20]	1.10 [1.04, 1.15]	1.11 [1.06, 1.17]
Q4	1.15 [1.11, 1.20]	1.12 [1.04, 1.21]	1.14 [1.07, 1.20]	1.12 [1.06, 1.18]
*Cumulative POA*				
Q1	1	1	1	1
Q2	1.08 [1.04, 1.13]	1.12 [1.03, 1.21]	1.14 [1.09, 1.19]	1.09 [1.01, 1.17]
Q3	1.15 [1.10, 1.21]	1.17 [1.06, 1.28]	1.19 [1.12, 1.27]	1.16 [1.08, 1.25]
Q4	1.19 [1.12, 1.26]	1.20 [1.09, 1.34]	1.17 [1.06, 1.28]	1.19 [1.10, 1.29]

y, year; IRR, incidence rate ratio; CI, confidence interval; AMI, acute myocardial infarction; PM_2.5_, fine particulate matter; NO_2_, nitrogen dioxide; EC, elemental carbon; POA, primary organic aerosols; Q1–Q4, quartile 1–4

^a^Adjusted for age, calendar year, occupational exposure to diesel exhaust, socioeconomic factors, including education and income, civil status, BMI, and smoking

^b^Adjusted for sex, calendar year, occupational exposure to diesel exhaust, socioeconomic factors, including education and income, civil status, BMI, and smoking

## Discussion

Results from this register-based cohort study showed an increased risk of incident AMI in association with cumulative exposure to PM_2.5_, NO_2_, EC, and POA with the strongest exposure-response association observed for PM_2.5_. Splines illustrated non-linear associations between cumulative exposure to the four air pollutants and AMI. When stratifying on age, we found an increased relative risk of AMI of comparable strength for both the younger and the older age group across all four exposures. We also observed that NO_2_, EC, and POA were associated with similar relative risks of AMI in both women and men, whereas PM_2.5_ showed a stronger relative risk among women. Regarding recent exposure, analyses showed that PM_2.5_ was the only indicator of air pollution significantly associated with incident AMI.

We found an association between exposure to PM_2.5_ and elevated risk of incident AMI, similar to findings in some recent studies, all of which had study populations larger than a million people (Alexeeff et al. 2023; Bai et al. 2019a, b; Poulsen et al. 2023; Wei et al. 2024). However, other studies did not report this association (Cramer et al. 2020; Downward et al. 2018; Lipsett et al. 2011; Ljungman et al. 2019; Miller et al. 2007; Puett et al. 2009). Among the latter, the study populations and number of incident cases of AMI were rather small, and four of the studies only investigated incident AMI among women (Cramer et al. 2020; Lipsett et al. 2011; Miller et al. 2007; Puett et al. 2009), who are known to have a lower incidence of AMI (Schulte and Mayrovitz 2023). In contrast, our study population includes 903,415 individuals and 35,511 cases of incident AMI. Therefore, a likely reason for the non-significant associations reported in these previous studies is insufficient statistical power.

A meta-analysis on long-term exposure to air pollution reported a lower risk estimate for NO_2_ compared to PM_2.5_ in relation to incident IHD (Cesaroni et al. 2014), whereas we have found IRRs of comparable strength for NO_2_ and PM_2.5_ (Table 2). In contrast to our findings of a clear exposure-response relationship between cumulative exposure to NO_2_ and incident AMI (Table 2), many previous studies have reported risk estimates close to 1 (Atkinson et al. 2013; Bai et al. 2019b; Beelen et al. 2014; Gan et al. 2011; Olaniyan et al. 2022). However, other studies have found trends similar to ours with increased risk of AMI in relation to NO_2_ (Grazuleviciene et al. 2004; Kim et al. 2017; Roswall et al. 2017). As for EC and POA, there are limited existing evidence of an association with incident AMI. Yang et al. (2020) found that POA was more strongly associated with IHD mortality than EC, and Poulsen et al. (2023) measured EC exposure as a 5-year average and did not find any indications of an association, which is in contrast to our findings (Table 2).

The majority of previous studies on air pollution have used an average exposure across a 1-, 3- or 5-year time-window as the exposure metric (Alexeeff et al. 2023; Bai et al. 2019a, b; Miller et al. 2007; Poulsen et al. 2023; Puett et al. 2009; Rosenlund et al. 2009; Wei et al. 2024). Evidence from a large Canadian cohort study suggests that longer exposure-windows are relevant when investigating air pollution, given that stronger associations were observed between PM_2.5_ and mortality when using an exposure measure spanning more years (Crouse et al. 2020). Only a few studies on air pollution have used a longer exposure window, such as a 10-year average or in one case a 23-year average (Cramer et al. 2020; Ljungman et al. 2019; Olaniyan et al. 2022; Roswall et al. 2017). In our study, exposure data were available for the study population from 1979 and onwards, covering nearly 40 years. In contrast to previous studies, we did not estimate a yearly average but used a cumulative exposure measure.

Cumulative exposure may be crucial when taking the time frame of atherosclerosis development into account. In general, the development of clinically manifest IHD due to atherosclerosis may take 40–50 years, which implies a long-term perspective on air pollution as a risk factor (Insull 2009). It is hypothesized that long-term exposure to particulate matter may play a role in atherogenesis through plaque progression via different mechanistic pathways (Brook et al. 2004). A key pathway includes a state of systemic chronic low-grade inflammation originating as an inflammatory spill-over effect arising in the lungs (Macchi et al. 2023). On the other hand, it must be acknowledged that our measure of recent exposure is rather crude, and effects of short-term acute exposures cannot be examined in this study.

Cumulated exposure to air pollution is higher among the oldest people in the study population, which in combination with higher prevalence of chronic disease might result in higher risk in the elderly. In the age-stratified analyses, however, we observed exposure-response associations of comparable strength for both the age group <55 years and ≥55 years across all four air pollutants (Table 3). Similar to our results, Alexeeff et al. (2023) also found an association with incident AMI for both the younger (18–64 years) and the older (≥64 years) age group. Wei et al. (2024) observed similar tendencies for the age groups when stratifying by age. However, only people aged ≥65 years were included in that study. In contrast, Poulsen et al. (2023) reported risk estimates close to 1 for the age group 50–70 years in relation to PM_2.5_, NO_2_ and EC exposure. Meanwhile the three exposures were significantly associated with incident AMI for the age group 80 + years. They did, however, not investigate the association in a population below 50 years of age. Our study population is younger than those in other cohort studies (Miller et al. 2007; Poulsen et al. 2023; Roswall et al. 2017; Wei et al. 2024), and so it is a key finding of this study, that the risk of AMI is also increased among the younger population exposed to higher levels of air pollution.

A stronger relative risk of incident AMI in association with PM_2.5_ was observed for women, whereas the IRRs were similar for women and men regarding NO_2_, EC, and POA (Table 2). Wei et al. (2024) also reported a stronger association between PM_2.5_ and AMI for women compared to men, while Alexeeff et al. (2023) found a similar relative risk for women and men regarding incident AMI, but a higher relative risk for women in relation to IHD mortality. These findings could indicate that women are more sensitive towards exposure to PM_2.5_. However, the absolute risk is much lower among women (Table S7), meaning that even a small increase in the absolute risk would result in a higher relative risk. Several other studies found approximately the same risk estimates for women and men regarding NO_2_ (Bai et al. 2019b; Poulsen et al. 2023; Rosenlund et al. 2009) and EC (Poulsen et al. 2023), comparable to our findings.

When adjusting for additional covariates, we generally observed a slight increase in risk estimates, most pronounced for Q4 across air pollutants. This pattern has also been reported in other studies (Bai et al. 2019a, b; Poulsen et al. 2023). This finding might to some extent be explained by considering, that individuals with higher socioeconomic status are perhaps also those most exposed to air pollution at the residential address, since they live in the largest cities (Raaschou-Nielsen et al. 2022). This hypothesis was confirmed when examining the distribution of PM_2.5_ and NO_2_ across education, while income showed a u-shaped pattern (Fig. S2). Furthermore, our results suggest that the impact of occupational exposure to DEE is already included within the other covariates, since the IRRs in model 4 did not change compared to model 3.

### Strengths and limitations

The major strengths of this study are the large sample size with 903,415 individuals covering almost 20 million person-years of follow-up, the nationwide prospective cohort design, the long exposure assessment period, covering almost 40 years, as well as the long follow-up period from 1996 to 2018, where cases of AMI were identified. Furthermore, the validated Danish population registries enabled linking information on air pollution at the residential address with the incidence of AMI, occupation, and additional covariates for each person.

Some limitations also need consideration. Road-traffic noise has been associated with higher risk of IHD (Selander et al. 2013; Vienneau et al. 2015), and is correlated with air pollution through road traffic (Cramer et al. 2020; Héritier et al. 2019), but information on noise exposure was not available in our study. According to our DAG, it is therefore possible that we might overestimate the association (Fig. S1). Two studies accounting for road traffic noise found that the excess risk of AMI decreased when adjusting for noise (Héritier et al. 2019; Roswall et al. 2017), while other studies found that the risk estimates remained approximately the same (Bai et al. 2019b; Cramer et al. 2020). Secondly, given the register-based nature of the study, it was not possible to adjust for individual lifestyle-factors such as diet, alcohol intake, physical activity, smoking and BMI. Therefore, we cannot exclude the possibility of residual confounding. However, according to our DAG it should not be necessary to adjust for health behavior (Fig. S1). We still accounted for smoking probability and BMI in the analyses, based on Danish JEMs where age, sex, job code and calendar year were considered, since there might be an unobserved confounder of air pollution and health behavior that we have not considered (Tennant et al. 2021). A Swedish JEM also allowed us to adjust for occupational DEE, which is a strength in the present study, and has not been adjusted for in previous studies. It allowed us to attempt to isolate the impact of residential exposure to air pollution.

Moreover, our study benefits from the advanced modelling of exposure at the residential address, covering all citizens in Denmark from 1979 and onwards, where the individual monthly exposure estimates constitute a high-resolution data foundation for the exposure assessment. Less than 1% of the addresses remained unidentified between 1979 and 2017. It was, however, not possible to account for non-residential exposure, such as occupational exposure to air pollution besides DEE, as well as exposure during commuting or other outdoor activities. A limitation of this study is therefore the potential exposure misclassification. Nonetheless, it is unlikely that the misclassification should be differentially distributed among individuals who developed AMI and those who did not; thus, most likely leading to underestimations of the association between air pollution and AMI. Furthermore, young adults may be more mobile and spend more time away from their registered residential addresses, which could result in less precise exposure estimates for this age group. This may introduce some degree of non-differential exposure misclassification, since the misclassification does not depend on the outcome. However, the study population was followed over a long period, and air pollution exposure was cumulated over time, which likely minimizes the impact of the potential non-differential exposure misclassification due to short-term mobility differences.

Another limitation of this study is the constraint in regression models, making it difficult to isolate the impact of each exposure on AMI, since the exposures are strongly correlated. In two-pollutant models it is largely arbitrary which component is showing a statistically significant association (Table S2). In our one-pollutant models it is possible that the identified associations between one of the four air pollutants and AMI are due to strong correlations with other air pollutants.

### Public health implications

Given that all individuals are exposed to air pollution, even small increases in risk of AMI on the individual level can have a large impact on the population level. Based on the estimated IRRs in Q2–Q4 of 1.08 [95% CI 1.04, 1.13], 1.14 [95% CI 1.09, 1.20] and 1.24 [95% CI 1.18, 1.31] in model 4 for PM2.5, approximately 10.3% of incident AMI cases may be attributed to higher exposure levels (Q2–Q4) compared to the lowest exposure group (Q1), based on population attributable risk calculations. This corresponds to 535 of the 5196 annual AMI cases in Denmark that could potentially be prevented if exposure levels were reduced to the level in Q1 (Hjerteforeningen 2023). The example highlights the public health impact of being exposed to higher levels of air pollution.

Furthermore, we would like to emphasize two aspects that may influence future policies. One, identifying the individual risk contributions of air pollution components are methodologically challenging due to their co-occurrence in complex mixtures originating from shared sources, such as fossil fuel combustion. This makes independent regulation of air pollutants difficult and isolating and removing a single pollutant component is not feasible in practice. Two, in this study, the focus has been on a younger study population; hence our work provides new insights and demonstrates that AMI due to air pollution exposure is not solely an issue among older individuals. Therefore, policies targeting population-wide exposure reduction in air pollution through regulation on emission are warranted and is essential for protecting public health at all ages.

## Conclusion

This study contributes to the existing evidence by showing that cumulative exposure to PM_2.5_, NO_2_, EC, and POA are associated with an increased risk of incident AMI with clear exposure-response relationships. These associations were also observed in the age group <55 years, indicating that the risk of serious disease due to air pollution is also present in the younger part of the population, further underlining the need for preventive efforts to lower air pollution levels.

## Supplementary Information

Below is the link to the electronic supplementary material.

Supplementary Material 1

## Data Availability

The data underlying the findings of this study are not openly available and is stored in a protected server environment hosted by Statistics Denmark.
