# Supplementary material for: Long-term exposure to ambient air pollution and risk of incident acute myocardial infarction in a nationwide register-based cohort study
Source: Int Arch Occup Environ Health. 2026 Jan 16;99(2):9. doi: 10.1007/s00420-025-02198-9 (PMC12811169; doi:10.1007/s00420-025-02198-9)
Supplement: Supplementary file 1 — Supplementary Material 1 [file 420_2025_2198_MOESM1_ESM.docx]

International Archives of Occupational and Environmental Health

Long-term exposure to ambient air pollution and risk of incident acute myocardial infarction in a nationwide register-based cohort study

Nikoline Leo Fleischer^1^, Esben Meulengracht Flachs^1^, Matthias Ketzel^2^, Jørgen Brandt^2^, Jibran Khan^2,3,4^, Per Gustavsson^5^, Ingrid Sivesind Mehlum^1,6,7^, Morten Böttcher^8,9^, Camilla Sandal Sejbæk^1^, Jens Peter Bonde^1^, Regitze Sølling Wils^1^

^1^ Department of Occupational and Environmental Medicine, Copenhagen University Hospital – Bispebjerg and Frederiksberg, Denmark

^2^ Department of Environmental Science, Aarhus University, Roskilde, Denmark

^3^ Danish Big Data Centre for Environment and Health (BERTHA), Aarhus University, Roskilde, Denmark

^4^ Department of Environment and Biological Sciences, University of Eastern Finland, Kuopio, Finland

^5^ Institute of Environmental Medicine, Karolinska Institutet, Stockholm, Sweden

^6^ Department of Public Health, University of Copenhagen, Copenhagen, Denmark

^7^ Department of Occupational Medicine and Epidemiology, National Institute of Occupational Health (STAMI), Oslo, Norway

^8^ Department of Cardiology, University Clinic for Cardiovascular Research, Gødstrup Hospital, Herning, Denmark

^9^ Department of Clinical Medicine, Aarhus University, Aarhus, Denmark

Supplemental material

**Figure S1.** Directed Acyclic Graph for residential air pollution (PM_2.5_, NO_2_, EC and POA) exposure and acute myocardial infarction (AMI).

**Table S1.** Characteristics of the DOC*X Dust Cohort (1996-2018) across PM_2.5_ quartiles (PY = 19,357,326).

**Table S2.** Correlation between the quartiles of PM_2.5_, NO_2_, EC and POA (cumulative) based on Spearman rank correlation coefficients.

**Table S3.** Associations between cumulative exposure (1979-2017) to PM_2.5_, NO_2_, EC and POA and incidence rate ratios for incident AMI with mutual adjustment for PM_2.5_ and NO_2_.

**Table S4.** Associations between cumulative exposure to PM_2.5_, NO_2_, EC and POA (per IQR exposure increment) and incidence rate ratios for incident AMI.

**Table S5.** Associations between recent exposure (the preceding year) to PM_2.5_, NO_2_, EC and POA and incidence rate ratios for incident AMI.

**Table S6.** Age- and sex-stratified analyses for recent exposure (the preceding year) to PM_2.5_, NO_2_, EC and POA and incidence rate ratio for incident AMI.

**Table S7**. Absolute risk per 1000 person-years for men, women, and different age groups.

**Figure S2.** Boxplots illustrating a) exposure to PM_2.5_ across education; b) exposure to PM_2.5_ across income quartiles; c) exposure to NO_2_ across education, and d) exposure to NO_2_ across income quartiles.


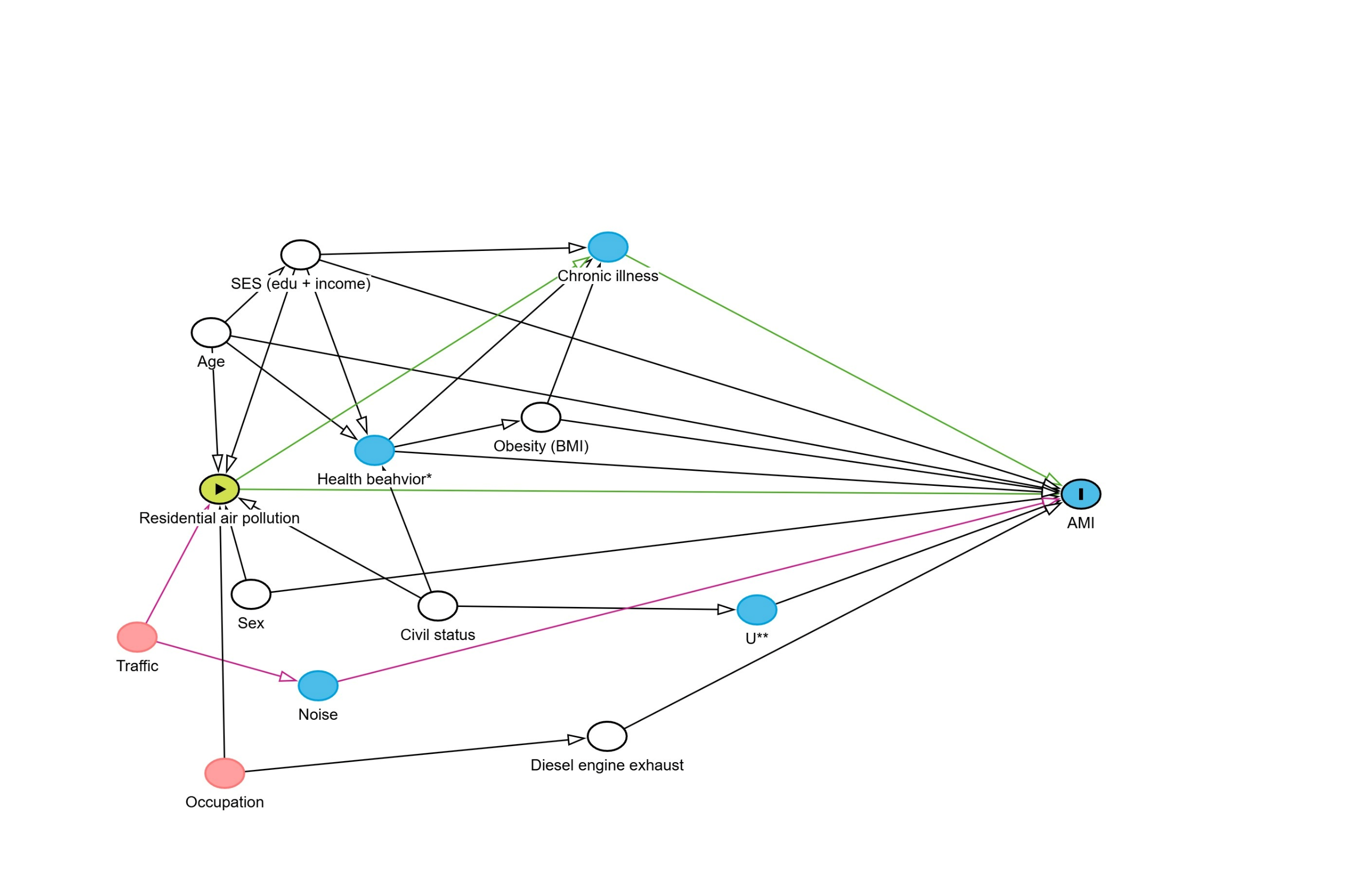


**Figure S1.** Directed Acyclic Graph for residential air pollution (PM_2.5_, NO_2_, EC and POA) exposure and acute myocardial infarction (AMI)

^*^Smoking, alcohol, physical activity, diet

^**^Unmeasured factor

**Table S1.** Characteristics of the DOC*X Dust Cohort (1996-2018) across PM_2.5_ quartiles (PY = 19,357,326)

| Characteristic |  | Q1  (194.6-340.9) |  | Q2  (>340.9-402.7) |  | Q3  (>402.7-464.2) |  | Q4  (>464.2-1,319.7) |
| --- | --- | --- | --- | --- | --- | --- | --- | --- |
|  |  | Total (%) |  | Total (%) |  | Total (%) |  | Total (%) |
| *PY* |  | 4,847,102 |  | 4,842,849 |  | 4,837,303 |  | 4,830,072 |
| Sex |  |  |  |  |  |  |  |  |
| Male |  | 2,433,800 (50.2) |  | 2,412,696 (49.8) |  | 2,376,426 (49.1) |  | 2,329,347 (48.2) |
| Female |  | 2,413,302 (49.8) |  | 2,430,153 (50.2) |  | 2,460,877 (50.9) |  | 2,500,725 (51.8) |
| Age |  |  |  |  |  |  |  |  |
| 35-54 |  | 4,603,933 (95.0) |  | 3,375,336 (69.7) |  | 1,822,732 (37.7) |  | 539,295 (11.2) |
| 55-73 |  | 243,169 (5.0) |  | 1,467,513 (30.3) |  | 3,014,571 (62.3) |  | 4,290,777 (88.8) |
| Highest education |  |  |  |  |  |  |  |  |
| Long |  | 250,606 (5.2) |  | 301,619 (6.2) |  | 330,401 (6.8) |  | 421,956 (8.7) |
| Medium |  | 839,906 (17.3) |  | 889,356 (18.4) |  | 921,289 (19.0) |  | 977,398 (20.2) |
| Short |  | 190,876 (3.9) |  | 203,293 (4.2) |  | 210,888 (4.4) |  | 213,300 (4.4) |
| Upper secondary |  | 2,240,488 (46.2) |  | 2,223,004 (45.9) |  | 2,214,994 (45.8) |  | 2,147,672 (44.5) |
| Lower secondary |  | 1,268,319 (26.2) |  | 1,171,360 (24.2) |  | 1,113,227 (23.0) |  | 1,027,944 (21.3) |
| Missing |  | 56,907 (1.2) |  | 54,217 (1.1) |  | 46,504 (1.0) |  | 41,802 (0.9) |
| Income |  |  |  |  |  |  |  |  |
| Q1 |  | 1,085,725 (22.4) |  | 1,161,891 (24.0) |  | 1,189,164 (24.6) |  | 1,225,671 (25.4) |
| Q2 |  | 1,215,651 (25.1) |  | 1,162,771 (24.0) |  | 1,150,240 (23.8) |  | 1,145,257 (23.7) |
| Q3 |  | 1,199,990 (24.7) |  | 1,183,669 (24.4) |  | 1,179,812 (24.4) |  | 1,115,375 (23.1) |
| Q4 |  | 1,144,068 (23.6) |  | 1,150,091 (23.8) |  | 1,169,542 (24.1) |  | 1,218,894 (25.2) |
| Missing |  | 201,668 (4.2) |  | 184,427 (3.8) |  | 148,545 (3.1) |  | 124,875 (2.6) |
| Civil status |  |  |  |  |  |  |  |  |
| Partner |  | 3,589,237 (74.0) |  | 3,499,807 (72.3) |  | 3,447,692 (71.3) |  | 3,248,305 (67.3) |
| No partner |  | 1,257,864 (26.0) |  | 1,343,042 (27.7) |  | 1,389,610 (28.7) |  | 1,581,768 (32.7) |
| BMI^a^ |  |  |  |  |  |  |  |  |
| 0- <1 years |  | 2,821,444 (58.2) |  | 2,303,241 (47.6) |  | 2,094,701 (43.3) |  | 2,017,215 (41.8) |
| 1-<9 years |  | 1,671,055 (34.5) |  | 1,578,954 (32.6) |  | 1,117,959 (23.1) |  | 801,096 (16.6) |
| 9-42 years |  | 354,603 (7.3) |  | 960,654 (19.8) |  | 1,624,643 (33.6) |  | 2,011,761 (41.6) |
| Smoking probability^a^ |  |  |  |  |  |  |  |  |
| 0-<6 years |  | 1,060,872 (21.9) |  | 1,083,934 (22.4) |  | 1,104,929 (22.8) |  | 1,148,742 (23.8) |
| 6-<10 years |  | 812,276 (16.8) |  | 824,870 (17.0) |  | 834,261 (17.3) |  | 857,579 (17.7) |
| 10-<15 years |  | 1,534,000 (31.6) |  | 1,519,086 (31.4) |  | 1,515,513 (31.3) |  | 1,507,158 (31.2) |
| 15-42 years |  | 1,439,954 (29.7) |  | 1,414,959 (29.2) |  | 1,382,600 (28.6) |  | 1,316,593 (27.3) |
| DEE |  |  |  |  |  |  |  |  |
| Ever |  | 1,531,001 (31.6) |  | 1,579,704 (32.6) |  | 1,604,997 (33.2) |  | 1,540,460 (31.9) |
| Never |  | 3,316,101 (68.4) |  | 3,263,145 (67.4) |  | 3,232,306 (66.8) |  | 3,289,612 (68.1) |

Note: PY, person-years; Q1-Q4, quartile 1-4; PM_2.5_, fine particulate matter; DEE, diesel engine exhaust.

^a^Specified as number of years above 75^th^ percentile (1976-2017).

**Table S2.** Correlation between the quartiles of PM_2.5_, NO_2_, EC and POA

(cumulative) based on Spearman rank correlation coefficients

|  |  | PM_2.5_ |  | NO_2_ |  | EC |  | POM |
| --- | --- | --- | --- | --- | --- | --- | --- | --- |
| PM_2.5_ |  | 1 |  |  |  |  |  |  |
| NO_2_ |  | 0.745 |  | 1 |  |  |  |  |
| EC |  | 0.682 |  | 0.894 |  | 1 |  |  |
| POA |  | 0.857 |  | 0.798 |  | 0.744 |  | 1 |

Note: PM_2.5_, fine particulate matter; NO_2_, nitrogen dioxide; EC, elemental carbon;

POA, primary organic aerosols.

**Table S3.** Associations between cumulative exposure (1979-2017) to PM_2.5_, NO_2_, EC and POA and incidence

rate ratios for incident AMI with mutual adjustment for PM_2.5_ and NO_2_

|  | Incident AMI | | | | | | | | |
| --- | --- | --- | --- | --- | --- | --- | --- | --- | --- |
|  |  | Model 1^a^ |  | Model 2^b^ |  | Model 3^c^ |  | Model 4^d^ |  |
| Exposure (µg/m^3^ y) |  | IRR (95% CI) |  | IRR (95% CI) |  | IRR (95% CI) |  | IRR (95% CI) |  |
| Cumulative PM_2.5_ |  |  |  |  |  |  |  |  |  |
| Q1 (194.6-340.9) |  | 1 |  | 1 |  | 1 |  | 1 |  |
| Q2 (>340.9-402.7) |  | 1.06 [1.01, 1.10] |  | 1.05 [1.00, 1.10] |  | 1.02 [0.98, 1.07] |  | 1.02 [0.98, 1.07] |  |
| Q3 (>402.7-464.2) |  | 1.08 [1.02, 1.14] |  | 1.06 [1.00, 1.13] |  | 1.03 [0.97, 1.09] |  | 1.03 [0.97, 1.09] |  |
| Q4 (>464.2-1,319.7) |  | 1.12 [1.05, 1.21] |  | 1.11 [1.03, 1.19] |  | 1.07 [0.99, 1.15] |  | 1.07 [0.99, 1.15] |  |
| Cumulative NO_2_ |  |  |  |  |  |  |  |  |  |
| Q1 (123.7-398.5) |  | 1 |  | 1 |  | 1 |  | 1 |  |
| Q2 (>398.5-505.2) |  | 1.04 [1.00, 1.08] |  | 1.05 [1.02, 1.09] |  | 1.08 [1.04, 1.12] |  | 1.08 [1.04, 1.12] |  |
| Q3 (>505.2-640.0) |  | 1.05 [1.01, 1.09] |  | 1.07 [1.03, 1.12] |  | 1.12 [1.07, 1.16] |  | 1.12 [1.07, 1.17] |  |
| Q4 (>640.0-2,551.3) |  | 1.06 [1.01, 1.11] |  | 1.10 [1.05, 1.15] |  | 1.16 [1.11, 1.22] |  | 1.17 [1.11, 1.22] |  |
| Recent PM_2.5_ |  |  |  |  |  |  |  |  |  |
| Q1 (4.9-9.1) |  | 1 |  | 1 |  | 1 |  | 1 |  |
| Q2 (>9.1-10.4) |  | 1.06 [1.03, 1.10] |  | 1.06 [1.03, 1.10] |  | 1.05 [1.02, 1.09] |  | 1.05 [1.02, 1.09] |  |
| Q3 (>10.4-11.8) |  | 1.07 [1.03, 1.12] |  | 1.07 [1.03, 1.12] |  | 1.06 [1.01, 1.11] |  | 1.06 [1.01, 1.11] |  |
| Q4 (>11.8-50.2) |  | 1.11 [1.05, 1.18] |  | 1.11 [1.05, 1.17] |  | 1.10 [1.04, 1.16] |  | 1.10 [1.04, 1.16] |  |
| Recent NO_2_ |  |  |  |  |  |  |  |  |  |
| Q1 (4.2-11.5) |  | 1 |  | 1 |  | 1 |  | 1 |  |
| Q2 (>11.5-14.5) |  | 0.99 [0.96, 1.02] |  | 1.00 [0.97, 1.03] |  | 1.02 [0.98, 1.05] |  | 1.02 [0.99, 1.05] |  |
| Q3 (>14.5-18.8) |  | 1.02 [0.98, 1.05] |  | 1.04 [1.00, 1.08] |  | 1.07 [1.04, 1.11] |  | 1.08 [1.04, 1.11] |  |
| Q4 (>18.8-80.0) |  | 1.04 [1.00, 1.08] |  | 1.07 [1.02, 1.11] |  | 1.10 [1.06, 1.15] |  | 1.11 [1.06, 1.15] |  |

Note: y, year; IRR, incidence rate ratio; CI, confidence interval; AMI, acute myocardial infarction; PM_2.5_, fine particulate matter;

NO_2_, nitrogen dioxide; EC, elemental carbon; POA, primary organic aerosols; Q1-Q4, quartile 1-4.

^a^Adjusted for age, sex, calendar year.

^b^Model 1 + additional adjusted for occupational exposure to diesel exhaust.

^c^Model 1 + additional adjusted for socioeconomic factors, including education and income, civil status, BMI, and smoking.

^d^Adjusted for all the above.

**Table S4.** Associations between cumulative exposure to PM_2.5_, NO_2_, EC and POA (per IQR exposure increment) and incidence rate ratios for incident AMI

|  |  |  | Incident AMI | | | | | | | | |
| --- | --- | --- | --- | --- | --- | --- | --- | --- | --- | --- | --- |
| Exposure |  | IQR |  | Model 1^a^ |  | Model 2^b^ |  | Model 3^c^ |  | Model 4^d^ |  |
| (µg/m^3^ y) |  | (µg/m3 y) |  | IRR (95% CI) |  | IRR (95% CI) |  | IRR (95% CI) |  | IRR (95% CI) |  |
| PM_2.5_ (1,944-15,836.6) |  | 1,478.4 |  | 1.11 [1.07, 1.14] |  | 1.13 [1.09, 1.16] |  | 1.15 [1.11, 1.18] |  | 1.15 [1.11, 1.18] |  |
| NO_2_ (1,484.1-30,615.6) |  | 2,889.2 |  | 1.04 [1.02, 1.06] |  | 1.05 [1.04, 1.07] |  | 1.07 [1.05, 1.09] |  | 1.07 [1.06, 1.09] |  |
| EC (72.4-9,791.4) |  | 134.8 |  | 1.01 [1.00, 1.03] |  | 1.02 [1.01, 1.04] |  | 1.03 [1.02, 1.04] |  | 1.03 [1.02, 1.04] |  |
| POA (154.2-1,134.0) |  | 213.1 |  | 1.01 [0.98, 1.03] |  | 1.04 [1.01, 1.07] |  | 1.10 [1.07, 1.13] |  | 1.10 [1.07, 1.13] |  |

Note: y, year; IQR, Inter Quartile Range; IRR, incidence rate ratio; CI, confidence interval; AMI, acute myocardial infarction; PM_2.5_, fine particulate matter; NO_2_, nitrogen dioxide; EC, elemental carbon; POA, primary organic aerosols.

^a^Adjusted for age, sex, calendar year.

^b^Model 1 + additional adjusted for occupational exposure to diesel exhaust.

^c^Model 1 + additional adjusted for socioeconomic factors, including education and income, civil status, BMI, and smoking.

^d^Adjusted for all the above.

**Table S5.** Associations between recent exposure (the preceding year) to PM_2.5_, NO_2_, EC and POA and incidence rate ratios for incident AMI

|  |  |  |  |  |  |  | Incident AMI | | | | | | | | |
| --- | --- | --- | --- | --- | --- | --- | --- | --- | --- | --- | --- | --- | --- | --- | --- |
|  |  |  |  |  |  |  |  | Model 1^a^ |  | Model 2^b^ |  | Model 3^c^ |  | Model 4^d^ |  |
| Exposure (µg/m^3^ y) |  | Mean |  | Person-years |  | Cases |  | IRR (95% CI) |  | IRR (95% CI) |  | IRR (95% CI) |  | IRR (95% CI) |  |
| Recent PM_2.5_^e^ |  |  |  |  |  |  |  |  |  |  |  |  |  |  |  |
| Q1 (4.9-9.1) |  | 8.1 |  | 4,814,621 |  | 11,520 |  | 1 |  | 1 |  | 1 |  | 1 |  |
| Q2 (>9.1-10.4) |  | 9.7 |  | 4,820,369 |  | 9,972 |  | 1.04 [1.01, 1.08] |  | 1.05 [1.02, 1.08] |  | 1.05 [1.02, 1.09] |  | 1.05 [1.02, 1.09] |  |
| Q3 (>10.4-11.8) |  | 11.1 |  | 4,825,659 |  | 7,693 |  | 1.05 [1.01, 1.10] |  | 1.06 [1.02, 1.11] |  | 1.07 [1.02, 1.12] |  | 1.07 [1.03, 1.12] |  |
| Q4 (>11.8-50.2) |  | 13.3 |  | 4,828,286 |  | 6,207 |  | 1.09 [1.04, 1.15] |  | 1.11 [1.05, 1.17] |  | 1.12 [1.07, 1.19] |  | 1.13 [1.07, 1.19] |  |
| Recent NO_2_^e^ |  |  |  |  |  |  |  |  |  |  |  |  |  |  |  |
| Q1 (4.2-11.4) |  | 9.4 |  | 4,818,025 |  | 10,813 |  | 1 |  | 1 |  | 1 |  | 1 |  |
| Q2 (>11.4-14.5) |  | 13.0 |  | 4,821,724 |  | 8,920 |  | 0.99 [0.96, 1.02] |  | 0.99 [0.96, 1.02] |  | 1.00 [0.97, 1.03] |  | 1.00 [0.97, 1.03] |  |
| Q3 (>14.5-18.8) |  | 16.5 |  | 4,824,052 |  | 8,272 |  | 1.01 [0.98, 1.05] |  | 1.03 [0.99, 1.06] |  | 1.04 [1.00, 1.08] |  | 1.04 [1.00, 1.08] |  |
| Q4 (>18.8-80.0) |  | 24.3 |  | 4,825,133 |  | 7,387 |  | 1.03 [0.98, 1.07] |  | 1.04 [0.99, 1.09] |  | 1.05 [1.00, 1.10] |  | 1.05 [1.00, 1.10] |  |
| Recent EC^e^ |  |  |  |  |  |  |  |  |  |  |  |  |  |  |  |
| Q1 (0.2-0.5) |  | 0.4 |  | 4,817,620 |  | 10,900 |  | 1 |  | 1 |  | 1 |  | 1 |  |
| Q2 (>0.5-0.6) |  | 0.6 |  | 4,821,526 |  | 9,037 |  | 0.96 [0.93, 0.99] |  | 0.97 [0.94, 1.00] |  | 0.98 [0.95, 1.01] |  | 0.98 [0.95, 1.01] |  |
| Q3 (>0.6-0.8) |  | 0.7 |  | 4,823,663 |  | 8,388 |  | 0.99 [0.96, 1.03] |  | 1.01 [0.97, 1.04] |  | 1.03 [0.99, 1.07] |  | 1.03 [0.99, 1.07] |  |
| Q4 (>0.8-34.9) |  | 1.1 |  | 4,826,125 |  | 7,067 |  | 0.98 [0.94, 1.03] |  | 1.00 [0.95, 1.04] |  | 1.01 [0.96, 1.06] |  | 1.01 [0.97, 1.06] |  |
| Recent POA^e^ |  |  |  |  |  |  |  |  |  |  |  |  |  |  |  |
| Q1 (0.5-1.2) |  | 1.1 |  | 4,819,991 |  | 9,779 |  | 1 |  | 1 |  | 1 |  | 1 |  |
| Q2 (>1.2-1.4) |  | 1.3 |  | 4,821,249 |  | 9,224 |  | 1.01 [0.98, 1.04] |  | 1.01 [0.98, 1.05] |  | 1.02 [0.99, 1.05] |  | 1.02 [0.99, 1.05] |  |
| Q3 (>1.4-1.7) |  | 1.5 |  | 4,822,872 |  | 8,537 |  | 1.00 [0.97, 1.04] |  | 1.01 [0.98, 1.05] |  | 1.03 [1.00, 1.07] |  | 1.03 [1.00, 1.07] |  |
| Q4 (>1.7-4.7) |  | 1.9 |  | 4,824,823 |  | 7,852 |  | 0.96 [0.92, 1.00] |  | 0.98 [0.94, 1.02] |  | 1.00 [0.96, 1.05] |  | 1.01 [0.96, 1.05] |  |

Note: y, year; IRR, incidence rate ratio; CI, confidence interval; AMI, acute myocardial infarction; PM_2.5_, fine particulate matter; NO_2_, nitrogen dioxide; EC, elemental carbon;

POA, primary organic aerosols; Q1-Q4, quartile 1-4.

^a^Adjusted for age, sex, calendar year.

^b^Model 1 + additional adjusted for occupational exposure to diesel exhaust.

^c^Model 1 + additional adjusted for socioeconomic factors, including education and income, civil status, BMI, and smoking.

^d^Adjusted for all the above.

^e^Exposure the preceding year; adjusted for cumulative exposure (quartile), lagged by 2 years.

**Table S6.** Age- and sex-stratified analyses for recent exposure (the preceding year) to PM_2.5_, NO_2_, EC and POA and incidence rate ratios for incident AMI (model 4)

|  | Incident AMI | | | | | | | | |
| --- | --- | --- | --- | --- | --- | --- | --- | --- | --- |
|  |  | Male^a^ |  | Female^a^ |  | Age < 55^b^ |  | Age ≥ 55^b^ |  |
| Exposure |  | IRR (95% CI) |  | IRR (95% CI) |  | IRR (95% CI) |  | IRR (95% CI) |  |
| Person-years (cases) |  | 9,515,659 (26,957) |  | 9,773,276 (8,435) |  | 10,306,190 (12,889) |  | 8,982,744 (22,503) |  |
| Recent PM_2.5_ |  |  |  |  |  |  |  |  |  |
| Q1 |  | 1 |  | 1 |  | 1 |  | 1 |  |
| Q2 |  | 1.04 [1.00, 1.08] |  | 1.11 [1.04, 1.18] |  | 1.17 [1.08, 1.27] |  | 1.04 [1.00, 1.08] |  |
| Q3 |  | 1.07 [1.02, 1.13] |  | 1.08 [0.99, 1.19] |  | 1.29 [1.18, 1.40] |  | 1.01 [0.95, 1.06] |  |
| Q4 |  | 1.12 [1.05, 1.19] |  | 1.16 [1.03, 1.29] |  | 1.31 [1.19, 1.44] |  | 1.08 [1.01, 1.17] |  |
| Recent NO_2_ |  |  |  |  |  |  |  |  |  |
| Q1 |  | 1 |  | 1 |  | 1 |  | 1 |  |
| Q2 |  | 1.00 [0.97, 1.04] |  | 0.98 [0.92, 1.05] |  | 1.10 [1.03, 1.17] |  | 0.98 [0.94, 1.02] |  |
| Q3 |  | 1.05 [1.01, 1.09] |  | 1.02 [0.95, 1.10] |  | 1.24 [1.16, 1.32] |  | 0.98 [0.94, 1.03] |  |
| Q4 |  | 1.04 [0.99, 1.10] |  | 1.07 [0.98, 1.18] |  | 1.27 [1.17, 1.37] |  | 0.99 [0.94, 1.06] |  |
| Recent EC |  |  |  |  |  |  |  |  |  |
| Q1 |  | 1 |  | 1 |  | 1 |  | 1 |  |
| Q2 |  | 0.97 [0.94, 1.01] |  | 1.01 [0.95, 1.07] |  | 1.06 [0.99;1.12] |  | 0.97 [0.93;1.01] |  |
| Q3 |  | 1.03 [0.99, 1.07] |  | 1.03 [0.96, 1.11] |  | 1.18 [1.11;1.26] |  | 0.99 [0.94;1.03] |  |
| Q4 |  | 1.01 [0.96, 1.06] |  | 1.02 [0.93, 1.12] |  | 1.17 [1.09;1.26] |  | 0.97 [0.91;1.03] |  |
| Recent POA |  |  |  |  |  |  |  |  |  |
| Q1 |  | 1 |  | 1 |  | 1 |  | 1 |  |
| Q2 |  | 1.01 [0.98, 1.05] |  | 1.04 [0.97, 1.11] |  | 1.05 [0.99, 1.11] |  | 1.00 [0.96, 1.04] |  |
| Q3 |  | 1.01 [0.97, 1.06] |  | 1.10 [1.02, 1.18] |  | 1.10 [1.03, 1.17] |  | 1.00 [0.95, 1.04] |  |
| Q4 |  | 1.00 [0.95, 1.05] |  | 1.03 [0.95, 1.12] |  | 1.09 [1.03, 1.17] |  | 0.95 [0.90, 1.00] |  |

Note: y, year; IRR, incidence rate ratio; CI, confidence interval; AMI, acute myocardial infarction; PM_2.5_, fine particulate matter; NO_2_, nitrogen dioxide; EC, elemental carbon; POA, primary organic aerosols; Q1-Q4, quartile 1-4.

^a^Adjusted for age, calendar year, occupational exposure to diesel exhaust, socioeconomic factors, including education and income,

marital status, BMI, and smoking.

^b^Adjusted for sex, calendar year, occupational exposure to diesel exhaust, socioeconomic factors, including education and income,

civil status, BMI, and smoking.

**Table S7.** Absolute risk per 1000 person-years for men, women, and different age groups

|  | Incident AMI | | | | | | | | |
| --- | --- | --- | --- | --- | --- | --- | --- | --- | --- |
|  |  | Men |  | Women |  | Age < 55 |  | Age ≥ 55 |  |
| Exposure |  | Per 1000 PY |  | Per 1000 PY |  | Per 1000 PY |  | Per 1000 PY |  |
|  |  | *2.83 [2.80, 2.87]* |  | *0.86 [0.84, 0.88]* |  | *1.25 [1.23, 1.27]* |  | *2.50 [2.47, 2.54]* |  |
| PM_2.5_ |  |  |  |  |  |  |  |  |  |
| Q1 |  | 1.78 [1.73, 1.83] |  | 0.41 [0.39, 0.44] |  | 1.05 [1.02, 1.08] |  | 2.12 [1.94, 2.31] |  |
| Q2 |  | 2.55 [2.49, 2.61] |  | 0.71 [0.68, 0.74] |  | 1.31 [1.28, 1.35] |  | 2.34 [2.27, 2.42] |  |
| Q3 |  | 3.22 [3.15, 3.29] |  | 1.02 [0.98, 1.06] |  | 1.51 [1.45, 1.56] |  | 2.46 [2.41, 2.52] |  |
| Q4 |  | 3.83 [3.75, 3.91] |  | 1.29 [1.25, 1.34] |  | 1.74 [1.63, 1.85] |  | 2.61 [2.56, 2.66] |  |
| NO_2_ |  |  |  |  |  |  |  |  |  |
| Q1 |  | 2.10 [2.04, 2.15] |  | 0.54 [0.51, 0.57] |  | 1.11 [1.08, 1.14] |  | 2.33 [2.23, 2.43] |  |
| Q2 |  | 2.73 [2.67, 2.80] |  | 0.79 [0.76, 0.83] |  | 1.24 [1.20, 1.28] |  | 2.49 [2.42, 2.56] |  |
| Q3 |  | 3.06 [2.99, 3.13] |  | 0.99 [0.95, 1.03] |  | 1.35 [1.30, 1.40] |  | 2.52 [2.46, 2.58] |  |
| Q4 |  | 3.47 [3.40, 3.55] |  | 1.11 [1.07, 1.16] |  | 1.52 [1.45, 1.58] |  | 2.55 [2.50, 2.61] |  |
| EC |  |  |  |  |  |  |  |  |  |
| Q1 |  | 2.15 [2.09, 2.21] |  | 0.57 [0.54, 0.60] |  | 1.12 [1.09, 1.16] |  | 2.37 [2.27, 2.47] |  |
| Q2 |  | 2.79 [2.73, 2.86] |  | 0.82 [0.79, 0.86] |  | 1.27 [1.23, 1.31] |  | 2.52 [2.45, 2.59] |  |
| Q3 |  | 3.10 [3.03, 3.17] |  | 0.98 [0.94, 1.02] |  | 1.33 [1.28, 1.38] |  | 2.53 [2.47, 2.59] |  |
| Q4 |  | 3.31 [3.229 3.37] |  | 1.07 [1.03, 1.11] |  | 1.43 [1.37, 1.49] |  | 2.51 [2.46, 2.57] |  |
| POA |  |  |  |  |  |  |  |  |  |
| Q1 |  | 1.84 [1.79, 1.89] |  | 0.45 [0.43, 0.48] |  | 1.07 [1.04, 1.10] |  | 2.26 [2.11, 2.42] |  |
| Q2 |  | 2.56 [2.50, 2.62] |  | 0.71 [0.68, 0.75] |  | 1.30 [1.26, 1.34] |  | 2.41 [2.33, 2.49] |  |
| Q3 |  | 3.31 [3.24, 3.38] |  | 1.01 [0.97, 1.05] |  | 1.49 [1.44, 1.55] |  | 2.54 [2.49, 2.60] |  |
| Q4 |  | 3.69 [3.61, 3.76] |  | 1.25 [1.20, 1.29] |  | 1.57 [1.47, 1.67] |  | 2.53 [2.48, 2.58] |  |

Note: PY, person-years; AMI, acute myocardial infarction; Q1-Q4, quartile 1-4


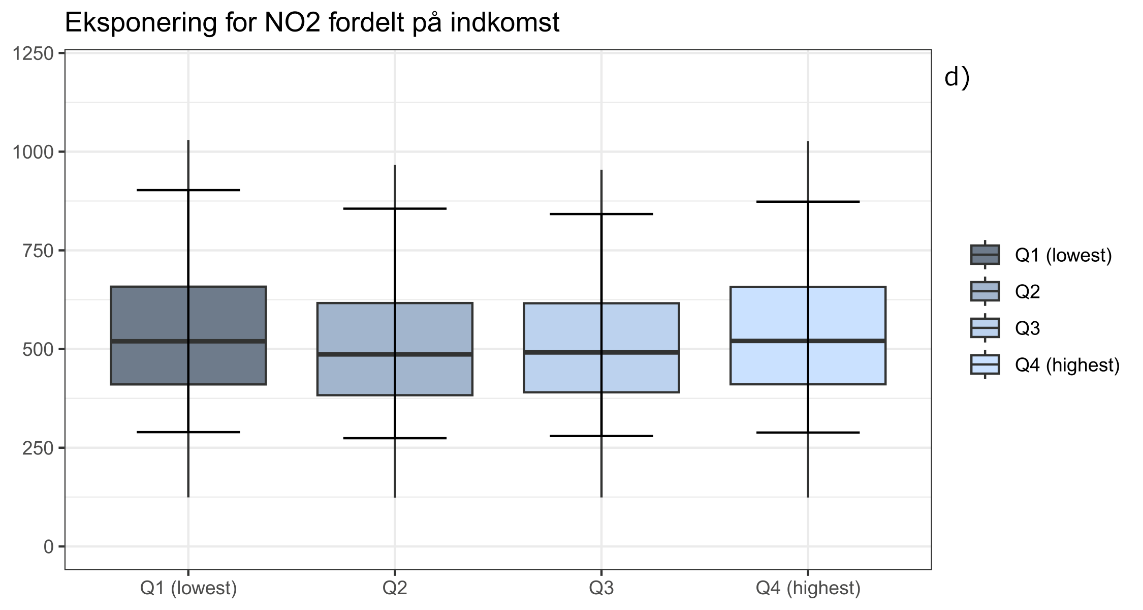

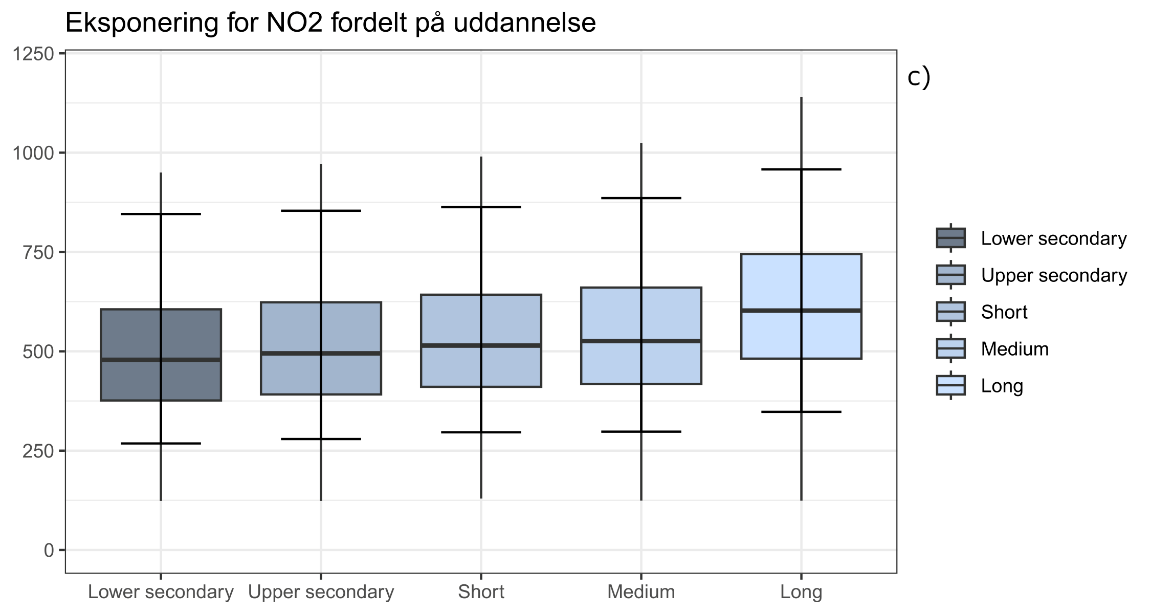

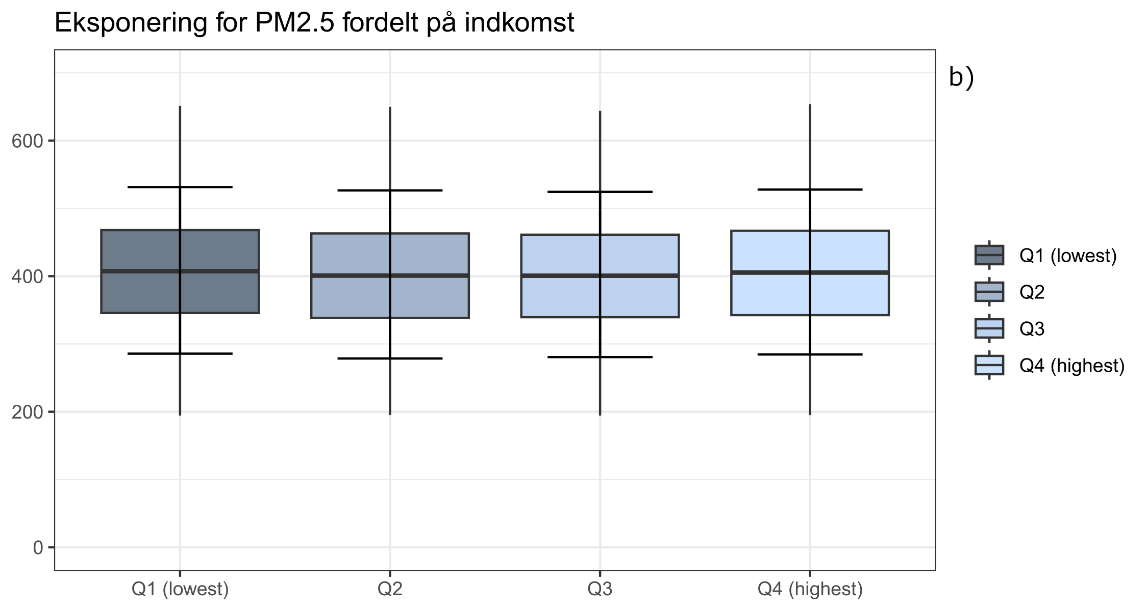

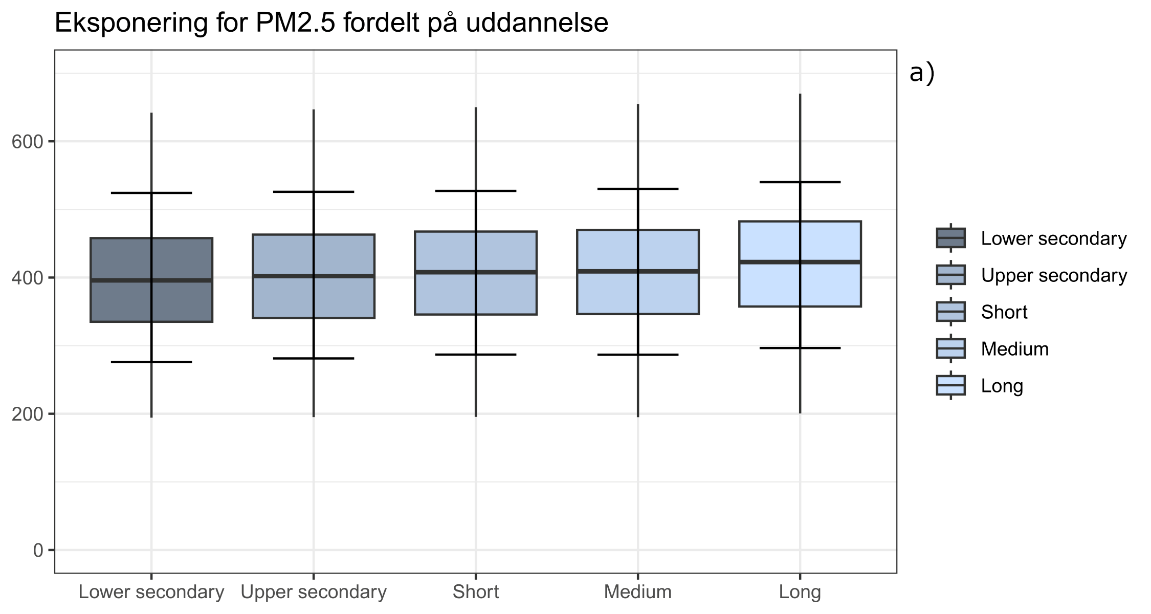


**Figure S2.** Boxplots illustrating a) exposure to PM_2.5_ across education; b) exposure to PM_2.5_ across income quartiles; c) exposure to NO_2_ across education; and d) exposure to NO_2_ across income quartiles.
